# BIM-mediated apoptosis and oncogene addiction

**DOI:** 10.18632/aging.101072

**Published:** 2016-09-29

**Authors:** Yulin Li, Anja Deutzmann, Dean W. Felsher

**Affiliations:** Division of Oncology, Departments of Medicine and Pathology, Stanford University, Stanford, CA 94305, USA

**Keywords:** BIM, apoptosis, oncogene addiction, oncogene inactivation, targeted therapy

Oncogene addiction is a phenomenon whereby suppression of a driver oncogene is associated with dramatic tumor regression that has been observed in experimental models and in response to targeted therapies [1]. However, the mechanism by which oncogene inactivation induces this massive reduction in tumor burden is not clear. In tumors addicted to the MYC oncogene, suppression of this oncogene leads to tumor regression that is associated with a marked increase in apoptosis. This at first glance appears to be paradoxical since generally oncogene activation, and MYC activation in particular, is associated with increased apoptosis. Recently, we have described a possible mechanism that may explain why inactivation of pro-apoptotic oncogenes, such as MYC, induce apoptosis [2].

To understand our recent results, we must first note that apoptosis is regulated by both pro-apoptotic and anti-apoptotic proteins. The pro-apoptotic protein, BIM (BCL2L11), is a BCL2 family member with three major isoforms (BIM-EL, BIM-L, and BIM-S) that can be generated from mRNA alternative splicing [3]. BIM works in concert with other pro-apoptotic proteins, such as PUMA, BAD, BAX, and anti-apoptotic proteins, such as BCL2, BCLXL, and MCL1, to regulate cell death and survival essential to normal tissue homeostasis. The precise regulation of BIM expression has been shown to be essential to normal development [3, 4]. Reduced BIM expression can disrupt normal development, induce autoimmunity and accelerate tumorigenesis [3, 5]. The relative dosage of BIM is critical, and its expression and activation is tightly regulated at many different levels, depending on the cellular context. BIM expression can be regulated transcriptionally by multiple transcriptional factors, posttrancriptionally by alternative splicing and microRNA binding, translationally by upstream open reading frames, posttranslationally by phosphorylation and degradation, as well as spatial localization and sequestration [6]. During normal physiological development, these regulatory mechanisms assure the precise control of BIM activation for tissue homeo-stasis. However, the same control mechanisms can also be perturbed by oncogenes that can contribute to tumorigenesis.

We found in multiple transgenic mouse models of oncogene (MYC, BCR-ABL, RAS) induced acute lymphocytic leukemia (ALL), that BIM was the key mediator of apoptosis observed upon oncogene inactivation [2]. In all of these mouse models, close examination of the expression of apoptotic regulatory proteins revealed that, BIM was the key regulator of the apoptosis that was observed. Importantly, at least two different mechanisms were involved, including a microRNA mediated mechanism but also a cell signaling based mechanism (Figure 1). Thus, just as BIM is physiologically regulated by multiple mechanisms, oncogenes appear to similarly co-opt different mechanisms to perturb BIM expression to regulate tumor survival, and abatement of expression of these oncogenes uncovered a unique vulnerability associated with increased apoptosis.

**Figure 1:**
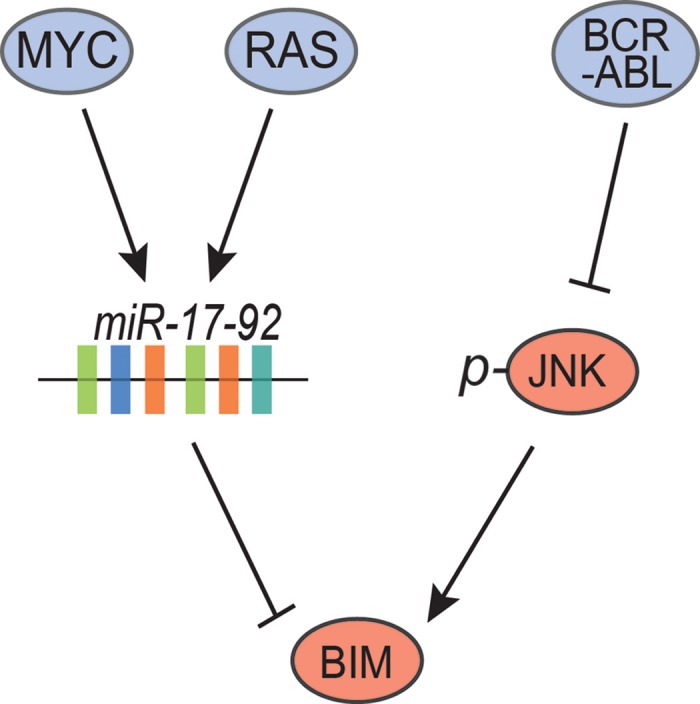
Inactivation of the MYC, RAS, and BCR-ABL oncogenes converges on BIM to induce apoptosis.

Importantly, our results are consistent with other reports that suggest that BIM is the key mediator of apoptosis induced by inactivation of the driver oncogene [7]. For example, BIM is induced to drive apoptosis in EGFR-dependent lung adenocarcinoma cells treated with tyrosine kinase inhibitors. In HER2-overexpressing human breast cancer cells and transgenic mouse breast cancer models, inactivation of HER2 upregulates BIM and suppression of BIM activity with ABT-737 have additive effects on tumor regression. Collectively, these observations also suggest that BIM is being actively suppressed by the driver oncogenes either directly or indirectly in order to block apoptosis. We speculate that BIM may be the universal apoptosis mediator to oncogene addiction. We expect that how a particular driver oncogene suppresses BIM will vary. Since BIM is an apoptosis regulator functional in many normal cell types, the driver oncogenes may simply hijack and rewire the control mechanisms of BIM expression that already exist in these cell types. Thus, BIM may be an important predictive biomarker as well as a direct mediator of the therapeutic efficacy of oncogene targeting agents.

Targeted therapy aimed at single oncogenes has not been successful for the treatment for most cancers. One potential solution is to further maximize the tumor cell killing by changing the pro-apoptotic versus anti-apoptotic balance. If BIM is the universal mediator of apoptosis upon oncogene inactivation, upregulation of BIM activity should synergize with targeted agents. In particular, BH3 mimetics, such as ABT-263 and obatoclax, can effectively activate BIM and tilt the balance towards apoptosis. Although the efficacy of BH3 mimetics is modest as single agents, they may potentiate the effects of targeted therapies as part of a rational combination. Thus, a combination of these two different classes of drugs should be tested on a broad scale and hold great potential for the treatment of many cancer types that exhibit oncogene addiction.

## References

[1] Weinstein IB (2002). Cancer. Addiction to oncogenes--the Achilles heal of cancer. Science.

[2] Li Y, Deutzmann A, Choi PS, Fan AC, Felsher DW (2016). BIM mediates oncogene inactivation-induced apoptosis in multiple transgenic mouse models of acute lymphoblastic leukemia. Oncotarget.

[3] Bouillet P, Metcalf D, Huang DC, Tarlinton DM, Kay TW, Köntgen F, Adams JM, Strasser A (1999). Proapoptotic Bcl-2 relative Bim required for certain apoptotic responses, leukocyte homeostasis, and to preclude autoimmunity. Science.

[4] Mailleux AA, Overholtzer M, Schmelzle T, Bouillet P, Strasser A, Brugge JS (2007). BIM regulates apoptosis during mammary ductal morphogenesis, and its absence reveals alternative cell death mechanisms. Dev Cell.

[5] Egle A, Harris AW, Bouillet P, Cory S (2004). Bim is a suppressor of Myc-induced mouse B cell leukemia. Proc Natl Acad Sci USA.

[6] Sionov RV, Vlahopoulos SA, Granot Z (2015). Regulation of Bim in Health and Disease. Oncotarget.

[7] Faber AC, Ebi H, Costa C, Engelman JA (2012). Apoptosis in targeted therapy responses: the role of BIM. Adv Pharmacol.

